# Redetermination of the crystal structure of arsenic tribromide, AsBr_3_

**DOI:** 10.1107/S241431462600235X

**Published:** 2026-03-11

**Authors:** Erik Uran, Kristian Radan, Matic Lozinšek

**Affiliations:** a Extreme Conditions Chemistry Laboratory (ECCL K2), Jožef Stefan Institute, Jamova cesta 39, 1000 Ljubljana, Slovenia; b Jožef Stefan International Postgraduate School, Jamova cesta 39, 1000 Ljubljana, Slovenia; Vienna University of Technology, Austria

**Keywords:** arsenic tribromide, pnictogen bonding, crystal structure, single-crystal X-ray diffraction

## Abstract

The crystal structure of AsBr_3_ was redetermined from low-temperature single-crystal X-ray diffraction data. Arsenic tribromide crystallizes in the ortho­rhom­bic Sohncke space group *P*2_1_2_1_2_1_ with *Z* = 4; the trigonal–pyramidal mol­ecule exhibits three symmetry-independent As—Br bond lengths [2.3386 (3)–2.3481 (3) Å]. An overview of the previous structure refinements of AsBr_3_ is provided, along with a comparison of the crystallographic parameters determined with higher precision in the current study.

## Abstract 2

The crystal structure of AsBr_3_ was redetermined by low-temperature single-crystal X-ray diffraction, leading to higher precision in the obtained bond lengths and angles.

## Graphical abstract

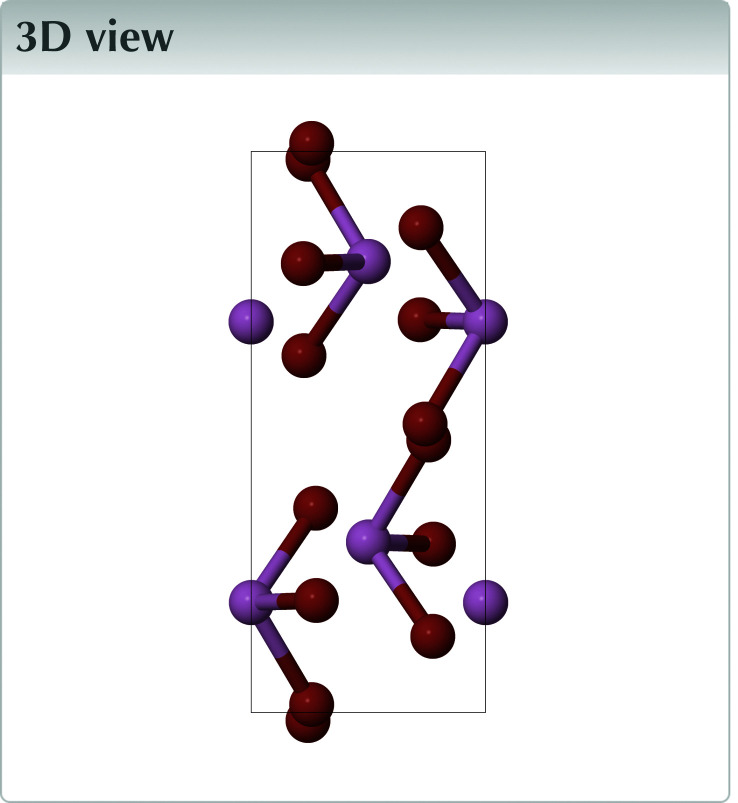

## Structure description

Pnictogen (Pn) elements (N, P, As, Sb, and Bi) form a wide range of halides, among which pnictogen(III) halides, PnX_3_, typically adopt a trigonal–pyramidal shape and crystallize predominantly as discrete mol­ecular units (Galy & Enjalbert, 1982 ▸). In general, mol­ecules of the PnX_3_ type are of inter­est owing to the presence of inter­molecular Pn⋯X contacts in their crystal structures, which arise from σ-holes located on the Pn atom (Varadwaj *et al.*, 2022 ▸). These features render them valuable model systems for studies of noncovalent inter­actions, particularly pnictogen bonding (Mahmudov *et al.*, 2020 ▸; Varadwaj *et al.*, 2022 ▸; Brammer *et al.*, 2023 ▸; Resnati *et al.*, 2024 ▸), as well as for high-pressure investigations, owing to their ability to expand their coordination sphere under high pressure (Schwarz *et al.*, 2019 ▸; Cai *et al.*, 2023 ▸; Gain *et al.*, 2024 ▸; Li *et al.*, 2025 ▸).

Among pnictogen(III) bromides, only the crystal structure of NBr_3_ (Klapötke, 1997 ▸) has not been determined, whereas PBr_3_ (Enjalbert & Galy, 1979*a* ▸), AsBr_3_ (Braekken, 1935 ▸; Singh & Swaminathan, 1964 ▸; Trotter, 1965 ▸; Singh & Swaminathan, 1967 ▸), SbBr_3_ (Cushen & Hulme, 1962 ▸, 1964 ▸), and BiBr_3_ (von Benda, 1980 ▸) have all been crystallographically characterized. Notably, BiBr_3_ is the only member reported to crystallize in both a mol­ecular form, composed of discrete trigonal–pyramidal BiBr_3_ mol­ecules, and a bromide-bridged polymeric form (von Benda, 1980 ▸). AsBr_3_ crystallizes in the *P*2_1_2_1_2_1_ space group and is isostructural with AsCl_3_ (Galy *et al.*, 2002 ▸) and the *α*-polymorph of SbBr_3_ (Cushen & Hulme, 1964 ▸). Its crystal structure differs from that of AsF_3_, which crystallizes in the *Pn*2_1_*a* space group (Enjalbert & Galy, 1979*b* ▸), and from AsI_3_, which adopts two polymorphs: the *R*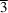 room-temperature form (Enjalbert & Galy, 1980 ▸) and the *P*3_2_12 high-temperature form (Galy & Enjalbert, 1982 ▸).

**Figure 1:**
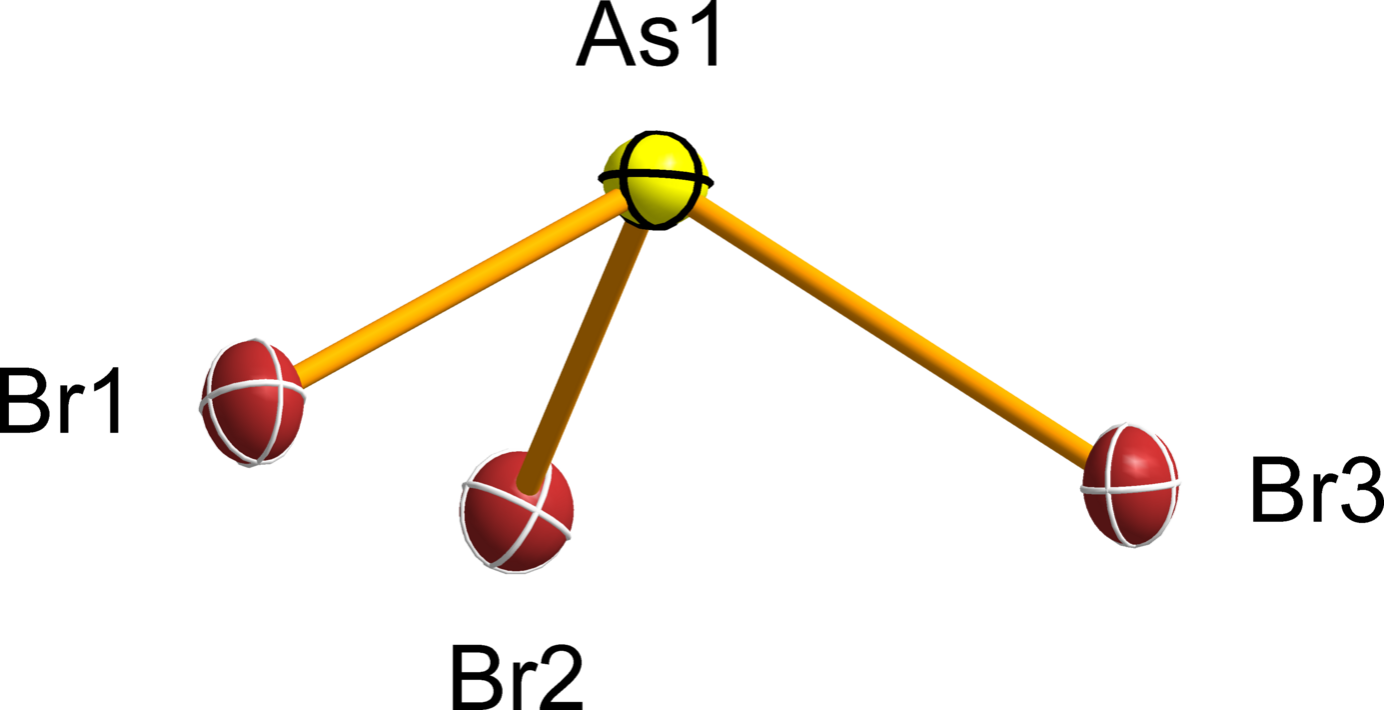
The mol­ecular structure of AsBr_3_ with displacement ellipsoids shown at the 50% probability level.

The AsBr_3_ crystal investigated at 100 K in the present work exhibits unit-cell parameters similar to those reported previously (Braekken, 1935 ▸; Singh & Swaminathan, 1964 ▸; Trotter, 1965 ▸; Singh & Swaminathan, 1967 ▸), but with significantly improved crystallographic parameters (Table 1 ▸). It crystallizes in the Sohncke space group *P*2_1_2_1_2_1_ and the unit cell contains four symmetrically equivalent AsBr_3_ mol­ecules adopting a slightly distorted *C*_3v_ geometry (true symmetry *C*_1_; Fig. 1 ▸).

The redetermined As—Br bond lengths and Br—As—Br angles are in good agreement with previously reported values (Table 1 ▸): As1—Br1 [2.3386 (3) Å], As1—Br2 [2.3416 (3) Å], and As1—Br3 [2.3481 (3) Å]; Br1—As1—Br2 [97.980 (11)°], Br1—As1—Br3 [98.124 (10)°], and Br2—As1—Br3 [99.460 (10)°]. The arsenic atom lies 1.1345 (3) Å above the trigonal plane defined by the three bromine atoms. Similar geometry was observed for cocrystallized AsBr_3_ mol­ecules with crystallographically imposed *C*_3v_ symmetry, with shorter As—Br bond lengths reported for the Br_3_As·C_6_Et_6_·AsBr_3_ cocrystal [2.322 (1) Å; 98.9 (1)°; 295 K] (Schmidbaur *et al.*, 1987 ▸) and longer for the (*p*-FC_6_H_4_)_3_P=Se·AsBr_3_ cocrystal [2.379 (3) Å; 96.54 (9)°; 100 K] (Alhanash *et al.*, 2012 ▸).

**Figure 2:**
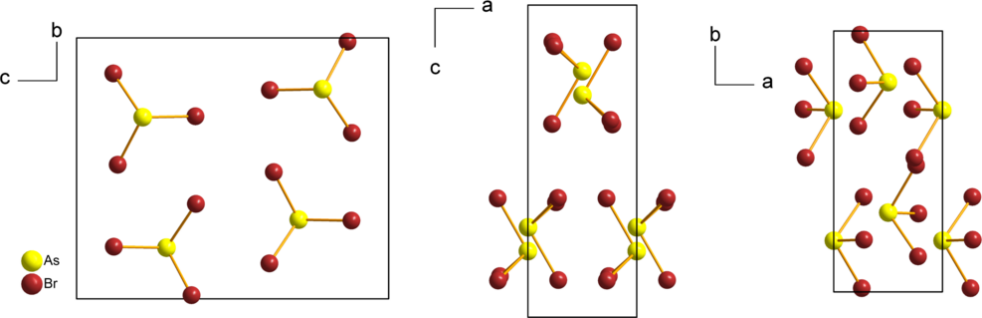
Unit cell and mol­ecular packing of AsBr_3_ viewed along the *a*, *b*, and *c* axes.

**Figure 3:**
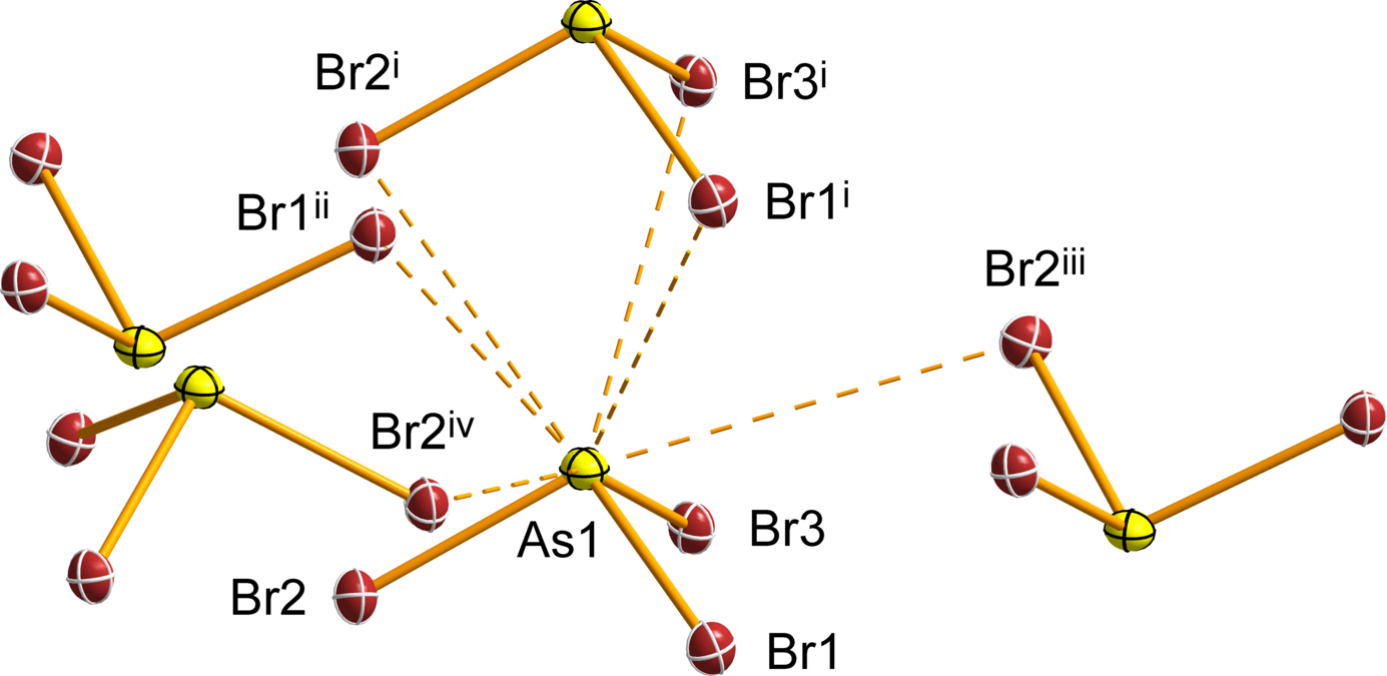
Inter­molecular As⋯Br contacts in AsBr_3_. Displacement ellipsoids are shown at the 50% probability level. [Symmetry codes: (i) −1 + *x*, *y*, *z*; (ii) 1 − *x*, −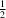 + *y*, 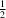 − *z*; (iii) 1 − *x*, 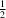 + *y*, 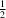 − *z*; (iv) −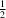 + *x*, 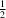 − *y*, 1 − *z*.]

The packing in the crystal structure of AsBr_3_ (Fig. 2 ▸) can be described as columns of AsBr_3_ mol­ecules stacked along the *a* axis, inter­connected to neighbouring columns *via* long As⋯Br and Br⋯Br contacts (Fig. 3 ▸, Table 2 ▸). The three shortest contacts, As1⋯Br3^i^ [3.6585 (3) Å], As1⋯Br2^i^ [3.6696 (3) Å], and As1⋯Br1^i^ [3.7507 (4) Å], involve neighbouring AsBr_3_ mol­ecules stacked above one another in a columnar arrangement along the *a* axis. The corresponding Br—As⋯Br angles deviate substanti­ally from linearity [130.199 (10)–132.418 (10)°]. Three additional As⋯Br contacts to AsBr_3_ mol­ecules in the adjacent columns are significantly longer than the sum of the van der Waals (vdW) radii [3.74 Å; Alvarez, 2013 ▸], and are nearly linear [3.8752 (3), 4.0974 (3), 4.1323 (4) Å; 168.505 (9), 166.766 (9), 162.03 (1)°] (Table 2 ▸). These inter­actions have been classified as pnictogen bonds (Varadwaj *et al.*, 2022 ▸). Br⋯Br contacts are likewise close to or longer than the sum of the vdW radii [3.72 Å], with only one contact being shorter [3.7044 (2) Å; Table 2 ▸]. Inter­molecular Br⋯Br distances of 3.7 Å have also been reported for AsBr_3_ in the liquid state (Hoge & Trotter, 1965 ▸).

## Synthesis and crystallization

Reactions were performed in fluorinated ethyl­ene propyl­ene (FEP) vessels as previously described (Uran & Lozinšek, 2025 ▸). AsBr_3_ formed as a side product during an attempt to synthesize CF_3_NH_3_[AsF_6_] from the reaction of BrCN with AsF_5_ and anhydrous HF (aHF), using a procedure similar to that reported previously (Baxter *et al.*, 2015 ▸). A 16 mg portion of the reaction product was recrystallized from aHF (0.37 ml) by cooling the solution to 213 K at an average rate of 5 K h^−1^. After crystallization, the volatiles were pumped off at 208 K, yielding crystals of AsBr_3_. A suitable crystal was selected using a low-temperature crystal mounting apparatus, as described previously (Lozinšek *et al.*, 2021 ▸; Motaln *et al.*, 2024 ▸), and mounted on the tip of a MiTeGen loop using Fomblin oil (Z25, SynQuest) (Motaln *et al.*, 2025 ▸).

## Refinement

Crystal data, data collection, and structure refinement details are summarized in Table 3 ▸.

## Supplementary Material

Crystal structure: contains datablock(s) I. DOI: 10.1107/S241431462600235X/wm4246sup1.cif

Structure factors: contains datablock(s) I. DOI: 10.1107/S241431462600235X/wm4246Isup2.hkl

CCDC reference: 2535582

Additional supporting information:  crystallographic information; 3D view; checkCIF report

## Figures and Tables

**Table 1:** Comparison of the crystallographic parameters for AsBr_3_ from previous structure determinations deposited in the Inorganic Crystal Structure Database (ICSD; Zagorac *et al.*, 2019 ▸) and the Cambridge Structural Database (CSD; Groom *et al.*, 2016 ▸) and from the present work

ICSD number/CSD deposition number	24589/1600611	24579/1600602	26774/1602157	24915/1600922	–/2535582
Reference	Braekken (1935 ▸)	Singh & Swaminathan (1964 ▸)	Trotter (1965 ▸)	Singh & Swaminathan (1967 ▸)	This work
Space Group	*P*2_1_2_1_2_1_	*P*2_1_2_1_2_1_	*P*2_1_2_1_2_1_	*P*2_1_2_1_2_1_	*P*2_1_2_1_2_1_
*a* (Å)	10.15	12.148	4.33 (1)	10.244	4.20575 (8)
*b* (Å)	12.07	10.244	10.24 (0.5)	12.148	10.08102 (18)
*c* (Å)	4.31	4.34	12.20 (0.5)	4.34	12.0632 (2)
*V* (Å^3^)	528.02	540.09	540.94	540.09	511.46 (2)
*T* (K)	*ns*	263	*ns*	*ns*	100
*R*	*ns*	0.23	0.188	0.143	0.0161
As—Br (Å)	2.26^‡^	2.325^‡^	2.354 (15)	2.349^‡^	2.3386 (3)
	2.32	2.335	2.354 (15)	2.352	2.3416 (3)
	2.41	2.354	2.384 (15)	2.383	2.3481 (3)
Br—As—Br (°)	97.0^‡^	99.47^‡^	97.3 (5)	96.43^‡^	97.980 (11)
	98.7	100.34	97.5 (5)	96.77	98.124 (10)
	101.2	100.78	98.2 (5)	99.05	99.460 (10)

*ns* not specified; ^‡^values calculated from atom coordinates reported in the ICSD.

**Table 2:** Inter­molecular As⋯Br and Br⋯Br contacts (Å, °) in the crystal structure of AsBr_3_

Contact	As⋯Br	Br—As⋯Br
(Br2)As1⋯Br3^i^	3.6585 (3)	132.217 (9)
(Br3)As1⋯Br2^i^	3.6696 (3)	132.418 (10)
(Br3)As1⋯Br1^i^	3.7507 (4)	130.199 (10)
(Br1)As1⋯Br1^ii^	3.8752 (3)	168.505 (9)
(Br2)As1⋯Br2^iii^	4.0974 (3)	166.766 (9)
(Br3)As1⋯Br2^iv^	4.1323 (4)	162.03 (1)
Contact	Br⋯Br	As—Br⋯Br
(As1)Br3⋯Br3^v^	3.7044 (2)	163.822 (11)
(As1)Br1⋯Br3^vi^	3.7213 (3)	140.565 (12)
(As1)Br1⋯Br3^vii^	3.7236 (3)	157.885 (11)
(As1)Br2⋯Br1^viii^	3.8321 (4)	150.861 (10)

Symmetry codes: (i) −1 + *x*, *y*, *z*; (ii) 1 − *x*, −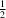 + *y*, 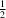 − *z*; (iii) 1 − *x*, 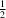 + *y*, 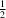 − *z*; (iv) −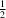 + *x*, 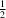 − *y*, 1 − *z*; (v) 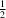 + *x*, 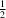 − *y*, −*z*; (vi) 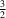 − *x*, 1 − *y*, 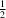 + *z*; (vii) 2 − *x*, 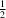 + *y*, 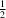 − *z*; (viii) 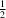 + *x*, 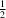 − *y*, 1 − *z*.

**Table 3:** Experimental details

Crystal data	
Chemical formula	AsBr_3_
*M*_r_	314.65
Crystal system, space group	Orthorhombic, *P*2_1_2_1_2_1_
Temperature (K)	100
*a*, *b*, *c* (Å)	4.20575 (8), 10.08102 (18), 12.0632 (2)
*V* (Å^3^)	511.46 (2)
*Z*	4
Radiation type	Ag *K*α, λ = 0.56087 Å
μ (mm^−1^)	15.94
Crystal size (mm)	0.15 × 0.06 × 0.03

Data collection	
Diffractometer	Rigaku OD, XtaLAB Synergy-S, Dualflex, Eiger2 R CdTe 1M
Absorption correction	Gaussian (*CrysAlis PRO*; Rigaku OD, 2025 ▸)
*T*_min_, *T*_max_	0.288, 0.963
No. of measured, independent and observed [*I* > 2σ(*I*)] reflections	30863, 2805, 2556
*R*_int_	0.041
(sin θ/λ)_max_ (Å^−1^)	0.890

Refinement	
*R*[*F*^2^ > 2σ(*F*^2^)], *wR*(*F*^2^), *S*	0.016, 0.028, 1.03
No. of reflections	2805
No. of parameters	39
Δρ_max_, Δρ_min_ (e Å^−3^)	0.43, −0.45
Absolute structure	Refined as an inversion twin
Absolute structure parameter	0.14 (6)

Computer programs: *CrysAlis PRO* (Rigaku OD, 2025 ▸), *OLEX2.solve* (Bourhis *et al.*, 2015 ▸), *SHELXL* (Sheldrick, 2015 ▸), *OLEX2* (Dolomanov *et al.*, 2009 ▸), *DIAMOND* (Brandenburg, 2022 ▸) and *publCIF* (Westrip, 2010 ▸).

## Data Availability

Data for this article are available from the Zenodo repos­itory at https://doi.org/10.5281/zenodo.18704110.

## full crystallographic data

### Arsenic tribromide Crystal data

**Table:**

AsBr_3_	*D*_x_ = 4.086 Mg m^−^^3^
*M**_r_* = 314.65	Ag *K*α radiation, λ = 0.56087 Å
Orthorhombic, *P*2_1_2_1_2_1_	Cell parameters from 20011 reflections
*a* = 4.20575 (8) Å	θ = 3.1–30.0°
*b* = 10.08102 (18) Å	µ = 15.94 mm^−^^1^
*c* = 12.0632 (2) Å	*T* = 100 K
*V* = 511.46 (2) Å^3^	Needle, clear colourless
*Z* = 4	0.15 × 0.06 × 0.03 mm
*F*(000) = 552	

### Arsenic tribromide Data collection

**Table:**

Rigaku OD, XtaLAB Synergy-S, Dualflex, Eiger2 R CdTe 1M diffractometer	2805 independent reflections
Radiation source: micro-focus sealed X-ray tube, PhotonJet (Ag) X-ray Source	2556 reflections with *I* > 2σ(*I*)
Mirror monochromator	*R*_int_ = 0.041
Detector resolution: 13.3333 pixels mm^-1^	θ_max_ = 30.0°, θ_min_ = 2.1°
ω scans	*h* = −7→6
Absorption correction: gaussian (CrysAlisPro; Rigaku OD, 2025)	*k* = −17→16
*T*_min_ = 0.288, *T*_max_ = 0.963	*l* = −21→20
30863 measured reflections	

### Arsenic tribromide Refinement

**Table:**

Refinement on *F*^2^	*w* = 1/[σ^2^(*F*_o_^2^) + (0.0097*P*)^2^] where *P* = (*F*_o_^2^ + 2*F*_c_^2^)/3
Least-squares matrix: full	(Δ/σ)_max_ = 0.001
*R*[*F*^2^ > 2σ(*F*^2^)] = 0.016	Δρ_max_ = 0.43 e Å^−^^3^
*wR*(*F*^2^) = 0.028	Δρ_min_ = −0.45 e Å^−^^3^
*S* = 1.03	Extinction correction: SHELXL (Sheldrick, 2015), Fc^*^=kFc[1+0.001xFc^2^λ^3^/sin(2θ)]^-1/4^
2805 reflections	Extinction coefficient: 0.0013 (3)
39 parameters	Absolute structure: Refined as an inversion twin
0 restraints	Absolute structure parameter: 0.14 (6)
Primary atom site location: iterative	

### Arsenic tribromide Special details

**Table:**

Geometry. All esds (except the esd in the dihedral angle between two l.s. planes) are estimated using the full covariance matrix. The cell esds are taken into account individually in the estimation of esds in distances, angles and torsion angles; correlations between esds in cell parameters are only used when they are defined by crystal symmetry. An approximate (isotropic) treatment of cell esds is used for estimating esds involving l.s. planes.
Refinement. Refined as a 2-component inversion twin.

### Arsenic tribromide Fractional atomic coordinates and isotropic or equivalent isotropic displacement parameters (Å^2^)

**Table:**

	*x*	*y*	*z*	*U*_iso_*/*U*_eq_	
As1	0.50086 (5)	0.30382 (2)	0.28814 (2)	0.01745 (4)	
Br1	0.75779 (7)	0.48585 (2)	0.36825 (2)	0.02074 (4)	
Br3	0.77836 (5)	0.30011 (2)	0.11926 (2)	0.01918 (4)	
Br2	0.77521 (5)	0.13600 (2)	0.38228 (2)	0.02066 (4)	

### Arsenic tribromide Atomic displacement parameters (Å^2^)

**Table:**

	*U*^11^	*U*^22^	*U*^33^	*U*^12^	*U*^13^	*U*^23^
As1	0.01404 (8)	0.02041 (8)	0.01790 (8)	−0.00019 (7)	0.00008 (7)	0.00075 (7)
Br1	0.02542 (9)	0.01809 (8)	0.01872 (8)	0.00129 (7)	−0.00113 (9)	−0.00212 (6)
Br3	0.02330 (8)	0.01976 (7)	0.01449 (7)	−0.00074 (8)	−0.00033 (7)	0.00048 (6)
Br2	0.02379 (9)	0.01909 (8)	0.01909 (8)	−0.00004 (7)	0.00067 (9)	0.00384 (6)

### Arsenic tribromide Geometric parameters (Å, º)

**Table:**

As1—Br1	2.3386 (3)	As1—Br2	2.3416 (3)
As1—Br3	2.3481 (3)		
			
Br1—As1—Br3	98.124 (10)	Br2—As1—Br3	99.460 (10)
Br1—As1—Br2	97.980 (11)		
